# Prostate Cancer Risk by *BRCA2* Genomic Regions

**DOI:** 10.1016/j.eururo.2020.05.005

**Published:** 2020-10

**Authors:** Tommy Nyberg, Debra Frost, Daniel Barrowdale, D. Gareth Evans, Elizabeth Bancroft, Julian Adlard, Munaza Ahmed, Julian Barwell, Angela F. Brady, Carole Brewer, Jackie Cook, Rosemarie Davidson, Alan Donaldson, Jacqueline Eason, Helen Gregory, Alex Henderson, Louise Izatt, M. John Kennedy, Claire Miller, Patrick J. Morrison, Alex Murray, Kai-Ren Ong, Mary Porteous, Caroline Pottinger, Mark T. Rogers, Lucy Side, Katie Snape, Vishakha Tripathi, Lisa Walker, Marc Tischkowitz, Rosalind Eeles, Douglas F. Easton, Antonis C. Antoniou

**Affiliations:** a Centre for Cancer Genetic Epidemiology, Department of Public Health and Primary Care, University of Cambridge, Cambridge, UK; b Manchester Regional Genetics Service, Central Manchester University Hospitals NHS Foundation Trust, Manchester, UK; c Oncogenetics Team, Division of Genetics and Epidemiology, The Institute of Cancer Research, London, UK; d Cancer Genetics Unit, Royal Marsden NHS Foundation Trust, London, UK; e Yorkshire Regional Genetics Service, Leeds Teaching Hospitals NHS Trust, Leeds, UK; f North East Thames Regional Genetics Service, Great Ormond Street Hospital for Children NHS Trust, London, UK; g Leicestershire Clinical Genetics Service, University Hospitals of Leicester NHS Trust, Leicester, UK; h North West Thames Regional Genetics Service, London North West University Healthcare NHS Trust, London, UK; i Peninsula Clinical Genetics Service, Royal Devon and Exeter NHS Foundation Trust, Exeter, UK; j North Trent Clinical Genetics Service, Sheffield Children’s NHS Foundation Trust, Sheffield, UK; k West of Scotland Regional Genetics Service, NHS Greater Glasgow and Clyde, Glasgow, UK; l South Western Regional Genetics Service, University Hospitals Bristol NHS Foundation Trust, Bristol, UK; m Nottingham Centre for Medical Genetics, Nottingham University Hospitals NHS Trust, Nottingham, UK; n North of Scotland Regional Genetics Service, NHS Grampian, Aberdeen, UK; o Northern Genetics Service, Newcastle upon Tyne Hospitals NHS Foundation Trust, Newcastle, UK; p South East Thames Regional Genetics Service, Guy’s and St Thomas’ NHS Foundation Trust, London, UK; q St James’s Hospital, Dublin, Republic of Ireland; r National Centre for Medical Genetics, Dublin, Republic of Ireland; s Merseyside and Cheshire Clinical Genetics Service, Liverpool Women’s NHS Foundation Trust, Liverpool, UK; t Northern Ireland Regional Genetics Service, Belfast Health and Social Care Trust, Belfast, UK; u Medical Genetics Services for Wales, Abertawe Bro Morgannwg University Health Board, Swansea, UK; v West Midlands Regional Genetics Service, Birmingham Women’s and Children’s NHS Foundation Trust, Birmingham, UK; w South East of Scotland Regional Genetics Service, NHS Lothian, Edinburgh, UK; x Medical Genetics Services for Wales, Betsi Cadwaladr University Health Board, Bodelwyddan, UK; y All Wales Medical Genetics Service, NHS Wales, Cardiff, UK; z Wessex Clinical Genetics Service, University Hospital Southampton NHS Foundation Trust, Southampton, UK; aa South West Thames Regional Genetics Service, St George’s University Hospitals NHS Foundation Trust, London, UK; bb Oxford Regional Genetics Service, Oxford University Hospitals NHS Foundation Trust, Oxford, UK; cc Department of Medical Genetics, National Institute for Health Research Cambridge Biomedical Research Centre, University of Cambridge, Cambridge, UK

**Keywords:** *BRCA2*, Genetic risk, Genomic region, Prospective cohort study, Prostate cancer, Prostate cancer cluster region

## Abstract

A *BRCA2* prostate cancer cluster region (PCCR) was recently proposed (c.7914 to 3′) wherein pathogenic variants (PVs) are associated with higher prostate cancer (PCa) risk than PVs elsewhere in the *BRCA2* gene. Using a prospective cohort study of 447 male *BRCA2* PV carriers recruited in the UK and Ireland from 1998 to 2016, we estimated standardised incidence ratios (SIRs) compared with population incidences and assessed variation in risk by PV location. Carriers of PVs in the PCCR had a PCa SIR of 8.33 (95% confidence interval [CI] 4.46–15.6) and were at a higher risk of PCa than carriers of other *BRCA2* PVs (SIR = 3.31, 95% CI 1.97–5.57; hazard ratio = 2.34, 95% CI 1.09–5.03). PCCR PV carriers had an estimated cumulative PCa risk of 44% (95% CI 23–72%) by the age of 75 yr and 78% (95% CI 54–94%) by the age of 85 yr. Our results corroborate the existence of a PCCR in *BRCA2* in a prospective cohort.

**Patient summary:**

In this report, we investigated whether the risk of prostate cancer for men with a harmful mutation in the *BRCA2* gene differs based on where in the gene the mutation is located. We found that men with mutations in one region of *BRCA2* had a higher risk of prostate cancer than men with mutations elsewhere in the gene.

## Take Home Message

*BRCA2* pathogenic variants located in a recently proposed prostate cancer cluster region confers higher risks of prostate cancer than other *BRCA2* variants. This report corroborates the existence of this prostate cancer cluster region in a large prospective cohort study.

We recently reported prostate cancer (PCa) risk estimates for pathogenic variants (PVs) in *BRCA2*, based on a prospective cohort of male carriers [1]. Variability in cancer risks due to genotype-phenotype correlations may allow for more individualised counselling and screening. We noted that PVs within the so-called ovarian cancer cluster region (OCCR) in exon 11 of the gene [2], [3], [4] were associated with a lower PCa risk than other *BRCA2* PVs [1], [3], [4]. PVs in the OCCR have consistently been shown to be associated with an increased ovarian cancer risk but a decreased breast cancer risk [2], [3], [5], [6], although the precise boundaries of the OCCR [3], [5] and the mechanisms behind this risk variation remain uncertain. It has been proposed that the likelihood that a PV triggers nonsense-mediated mRNA decay varies by genomic region [7], [8] so that OCCR PVs might produce a truncated or alternatively spliced protein the capability of which to suppress tumours varies by cancer type [2], [3], [5], [7], [8], but there is currently no experimental support for this hypothesis [7]. Shortly after the publication of our manuscript, Patel and coworkers [8] proposed the existence of a prostate cancer cluster region (PCCR) at the 3′ end of *BRCA2*, based on retrospective cohort data. This retrospective study reported that men with *BRCA2* PVs in the proposed PCCR have a higher risk of PCa (hazard ratio [HR] = 1.78, 95% confidence interval [CI] 1.25–2.52), particularly Gleason score ≥8 PCa (HR = 3.11, 95% CI 1.63–5.95), than men with PVs in the reference region c.1001 to c.7913, but did not present estimates of the absolute PCa risk for PCCR PV carriers [8]. In order to substantiate or refute this association, and to provide direct estimates of the absolute risk of PCa for carriers of *BRCA2* PCCR PVs, we have reanalysed our prospective data.

The prospective cohort comprised 447 male *BRCA2* PV carriers who were recruited to the EMBRACE study (http://ccge.medschl.cam.ac.uk/embrace/) through clinical genetics centres in the UK and Ireland between 1998 and 2016 at a median age of 51.4 yr (interquartile range 41.5–63.6 yr). The participants were counselled with regard to their PV. Detailed information on the cohort and on inclusion criteria, data collection, follow-up, and statistical analysis approach is available in our recent publication [1]. The participants’ PVs (listed in Supplementary Table S1) were grouped on the basis of position within the *BRCA2* gene, based on the proposed PCCR (c.7914 to 3′ [8]; HGVS nomenclature [http://varnomen.hgvs.org/]; using cDNA reference sequence NM_000059.3 and reference genome hg18) and the wide definition of the OCCR (c.2831 to c.6401) [1], [2], [3], [4]. We additionally considered the region bounded by c.756 and c.1000 in which Patel and coworkers [8] found evidence of an increased PCa risk, but due to a small sample size (*n* = 1) we could not estimate the PCa risk associated with this region. Here, we also present floating absolute risks (FARs) [9] to enable risk comparisons between any of the considered genomic regions.

The Anglia and Oxford Medical Research and Ethics Committee approved the study. All participants provided written informed consent.

**Fig. 1:**
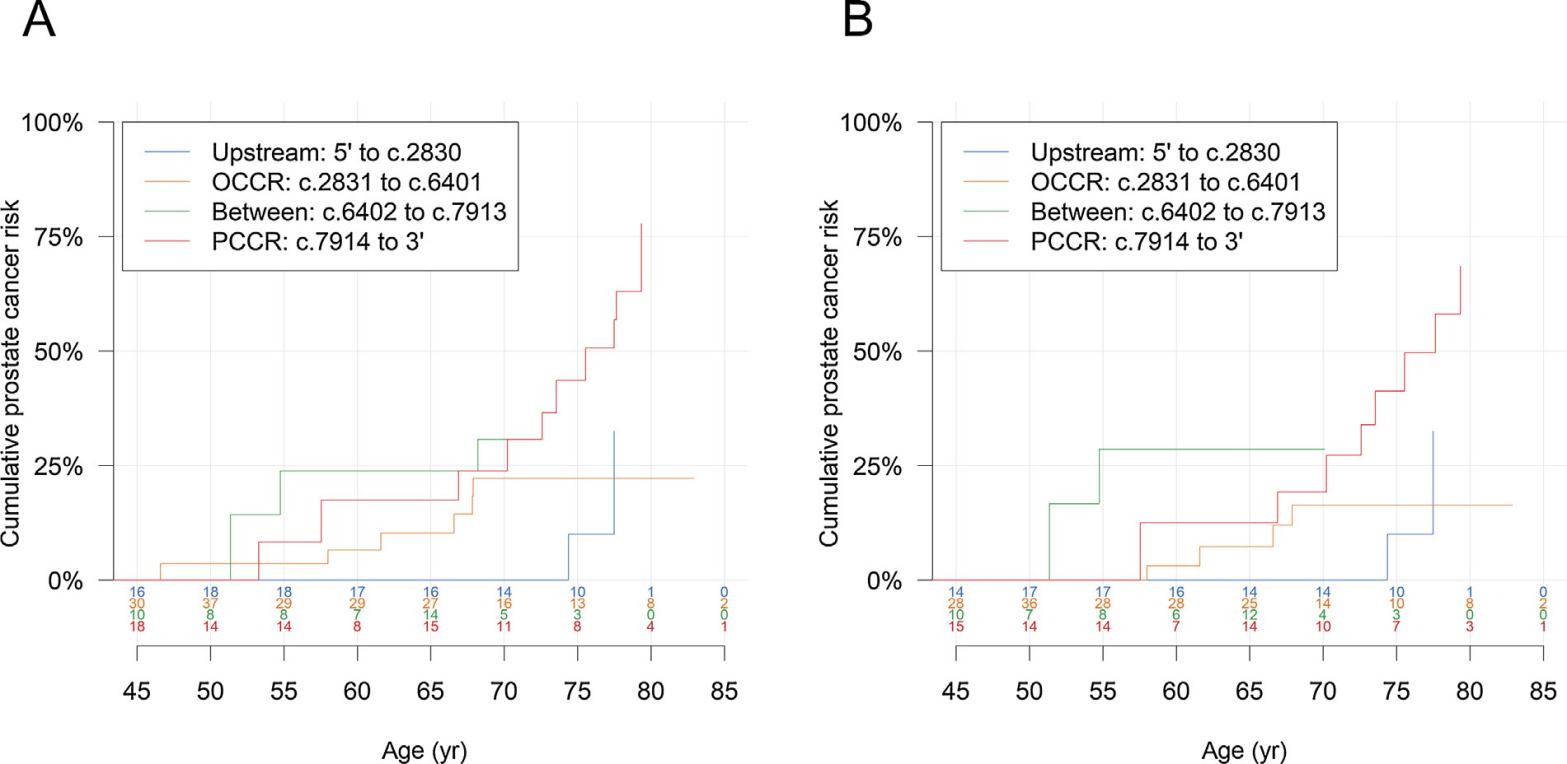
Absolute prostate cancer risk (A) by location of *BRCA2* pathogenic variant and (B) by location of *BRCA2* pathogenic variant and with follow-up initiated 6 mo after study entry. The number at risk at each age is shown above the *x*-axis. The curves are truncated at ages when fewer than five participants are at risk. OCCR = ovarian cancer cluster region; PCCR = prostate cancer cluster region.

Twenty-six participants developed PCa during a median follow-up of 5.3 yr (interquartile range 2.6–8.9 yr) [1]. Carriers of PVs in the PCCR (*n* = 93) had a PCa standardised incidence ratio (SIR) of 8.33 (95% CI 4.46–15.6), whereas carriers of PVs elsewhere in *BRCA2* (*n* = 354) had an SIR of 3.31 (95% CI 1.97–5.57) compared with population incidences. This corresponds to a significantly higher PCa risk associated with PVs in the PCCR than PVs not in the PCCR (HR = 2.34, 95% CI 1.09–5.03; Table 1). Compared with PVs in the region c.1001 to c.7913 [8], PCCR PVs were associated with an HR of 2.09 (95% CI 0.98–4.45). As previously reported, the SIR for carriers of PVs in the wide definition of the OCCR (*n* = 178) was 2.46 (95% CI 1.07–5.64) [1], and the risk for carriers of PCCR PVs was also significantly higher than that for OCCR PV carriers (HR = 3.41, 95% CI 1.27–9.16). The SIR for PVs located in the region bounded by the OCCR and the PCCR (c.6402 to c.7913; *n* = 66) was estimated to be 6.14 (95% CI 2.18–17.3), and the SIR for *BRCA2* PVs upstream of the OCCR (5′ to c.2830; *n* = 108) was 3.50 (95% CI 1.48–8.26). The FARs for the comparison of risks across the four regions suggested that the observed increased risk associated with PVs in the PCCR may partly be driven by the lower risk associated with PVs in the OCCR (Table 1). The proportional hazard assumption was violated for the model with all genomic regions fitted (Schoenfeld residual test, *p* =  0.003); in line with this the corresponding Kaplan-Meier plot indicated that the risks might be similar between OCCR and PCCR PV carriers at younger ages but deviate at older ages. PCCR PV carriers had an estimated cumulative PCa risk of 44% (95% CI 23–72%) by the age of 75 yr and 78% (95% CI 54–94%) by 85 yr. After omitting the first 6 mo of follow-up to assess the possible effect of screening-associated diagnoses of indolent PCa, the corresponding estimates were 41% (95% CI 20–73%) and 69% (95% CI 42–91%), respectively (Fig. 1).

**Table 1:** Prostate cancer risk by location of *BRCA2* pathogenic variant.

PV location	*N*	Person years	Observed events	Incidence rate per 1000 person years (95% CI)	Expected events	SIR (95% CI)	HR (95% CI)	FAR (95% CI)
*Compared with non-PCCR PVs*								
Non-PCCR (5′ to c.7913)	354	2029.8	15	7.39 (4.45–12.3)	4.53	3.31 (1.97–5.57)	Reference	
PCCR (c.7914 to 3′)	93	524.6	11	21.0 (11.4–38.7)	1.32	8.33 (4.46–15.6)	2.34 (1.09–5.03)	
*Compared with OCCR PVs*								
5′ to c.2830	108	625.8	5	7.99 (3.37–19.0)	1.43	3.50 (1.48–8.26)	1.72 (0.50–5.94)	1.72 (0.70–4.24)
OCCR (c.2831 to c.6401) a	178	1054.4	6	5.69 (2.54–12.8)	2.44	2.46 (1.07–5.64)	Reference	1.00 (0.43–2.33)
c.6402 to c.7913	66	338.8	4	11.8 (4.29–32.5)	0.65	6.14 (2.18–17.3)	3.23 (0.79–13.2)	3.23 (1.15–9.11)
PCCR (c.7914 to 3′)	93	524.6	11	21.0 (11.4–38.7)	1.32	8.33 (4.46–15.6)	3.41 (1.27–9.16)	3.41 (1.96–5.95)
Indeterminable	2							

CI = confidence interval; FAR = floating absolute risk; HR = hazard ratio; OCCR = ovarian cancer cluster region; PCCR = prostate cancer cluster region; PV = pathogenic variant; SIR = standardised incidence ratio.

a Detailed results for carriers of PVs in the OCCR are available in a previous publication [1].

The difference in PCa risk for PVs in the PCCR versus that in the OCCR remained statistically significant after adjusting for family history of PCa (number of first- and second-degree relatives diagnosed with PCa; adjusted HR = 3.00, 95% CI 1.06–8.54) or geographical location (adjusted HR = 3.79, 95% CI 1.41–10.2). This difference remained similar after omitting the first 6 mo of follow-up (HR = 3.96, 95% CI 1.18–13.3), related individuals (HR = 4.29, 95% CI 1.30–14.2), and carriers of PVs in the region c.756 to c.1000 (HR = 3.42, 95% CI 1.27–9.18) or missense variants (HR = 3.76, 95% CI 1.36–10.4). When carriers of the Ashkenazi founder PV c.5946delT (*n* = 42), which is located in the OCCR, was omitted, the difference in PCa risk between PCCR and OCCR PV carriers was not statistically significant, but the HR estimate was of similar magnitude (HR = 2.89, 95% CI 0.98–8.53; Supplementary Table S2).

We did not observe a higher risk of Gleason score ≥8 PCa for PVs in the PCCR than for PVs not in the PCCR (HR = 0.87, 95% CI 0.12–6.34) or in the region c.1001 to c.7913 (HR = 0.79, 95% CI 0.11–5.69). However, the HRs did not differ significantly from those for Gleason score ≤7 PCa (PCCR vs non-PCCR: HR = 3.32, 95% CI 1.25–8.84; test for heterogeneity, *p* =  0.052; PCCR vs c.1001 to c.7913: HR = 2.94, 95% CI 1.11–7.80; test for heterogeneity, *p* =  0.088).

Our results corroborate the observation that carriers of PVs in the PCCR of the *BRCA2* gene [8] are at a higher risk of PCa than other *BRCA2* PV carriers. Patel and coworkers [8] reported an HR of 1.78 (95% CI 1.25–2.52) compared with PVs in the region c.1001 to c.7913, consistent with our HR estimate of 2.09 (95% CI 0.98–4.45). Our findings do not support a stronger association with a more aggressive phenotype, but these estimates were based on a small number of cases and the associated CIs are wide. PV carriers may receive enhanced screening, which may lead to biases in comparisons against the population incidence [1]. However, current screening practices do not differ by *BRCA2* PV location, and so this is unlikely to have confounded the comparisons between the *BRCA2* genomic regions. A much larger cohort of unaffected carriers with longer follow-up is required to provide more precise PV-specific risk estimates and to further clarify whether the observed variation in risk reflects lower risks associated with PVs outside the OCCR and PCCR than the risk associated with PCCR PVs, or solely a lower risk associated with PVs in the OCCR.

  ***Author contributions*:** Tommy Nyberg had full access to all the data in the study and takes responsibility for the integrity of the data and the accuracy of the data analysis.

*Study concept and design*: Antoniou, Nyberg, Easton.

*Acquisition of data*: Frost, Barrowdale, Bancroft, Easton, Eeles, Evans, Tischkowitz, Adlard, Ahmed, Barwell, Brady, Brewer, Cook, Davidson, Donaldson, Eason, Gregory, Henderson, Izatt, Kennedy, Miller, Morrison, Murray, Ong, Porteous, Pottinger, Rogers, Side, Snape, Tripathi, Walker.

*Analysis and interpretation of data*: Nyberg, Antoniou, Easton.

*Drafting of the manuscript*: Nyberg, Antoniou, Easton.

*Critical revision of the manuscript for important intellectual content*: Evans, Frost, Barrowdale, Bancroft, Eeles, Tischkowitz, Adlard, Ahmed, Barwell, Brady, Brewer, Cook, Davidson, Donaldson, Eason, Gregory, Henderson, Izatt, Kennedy, Miller, Morrison, Murray, Ong, Porteous, Pottinger, Rogers, Side, Snape, Tripathi, Walker.

*Statistical analysis*: Nyberg, Antoniou, Easton.

*Obtaining funding*: Easton, Antoniou, Eeles, Evans.

*Administrative, technical, or material support*: Frost, Barrowdale, Bancroft.

*Supervision*: Antoniou, Tischkowitz.

*Other*: None.

  ***Financial disclosures:*** Tommy Nyberg certifies that all conflicts of interest, including specific financial interests and relationships and affiliations relevant to the subject matter or materials discussed in the manuscript (eg, employment/affiliation, grants or funding, consultancies, honoraria, stock ownership or options, expert testimony, royalties, or patents filed, received, or pending), are the following: None.

  ***Funding/Support and role of the sponsor*:** This work was supported by 10.13039/501100000289Cancer Research UK grants C12292/A20861 and C12292/A22820. EMBRACE was supported by Cancer Research UK grants C1287/A23382 and C1287/A26886. D. Gareth Evans is supported by a 10.13039/501100000272National Institute for Health Research grant to the Biomedical Research Centre, Manchester (IS-BRC-1215-20007). Rosalind Eeles is supported by Cancer Research UK grant C5047/A8385, and by National Institute for Health Research support to the Biomedical Research Centre at The Institute of Cancer Research and The Royal Marsden NHS Foundation Trust.

  ***Acknowledgements*:** We thank all the participants in the EMBRACE study. The data used in the analysis are available to other researchers upon request to the EMBRACE study coordinators (https://ccge.medschl.cam.ac.uk/embrace/).
