# Supplementary material for: Prostate Cancer Risk by BRCA2 Genomic Regions
Source: Eur Urol. 2020 Oct;78(4):494–7. doi: 10.1016/j.eururo.2020.05.005 (PMC7532700; doi:10.1016/j.eururo.2020.05.005)
Supplement: Supplementary file 1 [file mmc1.pdf]

Supplementary Table S1 – List of *BRCA2* pathogenic variants carried by the 447 study participants

Genomic level	Protein level	*n*
c.-227-?_67+?del	p.0	6
c.-227-?_6841+?del	p.0	1
c.17_18del	p.Lys6fs	1
c.22_23del	p.Arg8fs	2
c.26del	p.Pro9fs	5
c.36dup	p.Glu13*	1
c.104_110del	p.Leu35fs	2
c.314T>G	p.Leu105*	1
c.396T>A	p.Cys132*	1
c.407del	p.Asn136fs	2
c.470_474del	p.Lys157fs	1
c.516+1G>T	S	1
c.517-2A>G	S	2
c.538_539dup	p.Ser181fs	2
c.574_575del	p.Met192fs	3
c.631+1G>A	S	1
c.631+2T>G	p.Gly173fs	5
c.658_659del	p.Val220fs	4 [a]
c.755_758del	p.Asp252fs	19
c.765_770delinsAAACAAT	p.Asn255fs	1
c.1097T>G	p.Leu366*	1
c.1189_1190insTTAG	p.Gln397fs	7
c.1231del	p.Ile411fs	1
c.1310_1313del	p.Lys437fs	3
c.1654del	p.Ser552fs	1
c.1689G>A	p.Trp563*	3
c.1787_1799del	p.Asp596fs	2
c.1813del	p.Ile605fs	3
c.1813dup	p.Ile605fs	4
c.1889del	p.Thr630fs	1
c.1929del	p.Arg645fs	7
c.2409T>G	p.Tyr803*	3
c.2606C>G	p.Ser869*	1
c.2701del	p.Ala902fs	1
c.2760del	p.Ile921fs	1
c.2808_2811del	p.Ala938fs	10
c.2870del	p.Asn957fs	1
c.3009_3010del	p.His1003fs	1
c.3158T>G	p.Leu1053*	2
c.3195_3198del	p.Asn1066fs	2
c.3405C>A	p.Tyr1135*	1
c.3530_3533del	p.Asp1177fs	1

c.3545_3546del	p.Phe1182*	2
c.3599_3600del	p.Cys1200*	1
c.3680_3681del	p.Leu1227fs	1
c.3785C>G	p.Ser1262*	4
c.3847_3848del	p.Val1283fs	4
c.3860del	p.Asn1287fs	1
c.4037_4038del	p.Thr1346fs	1
c.4101del	p.Lys1367fs	1
c.4137_4141del	p.Ile1380fs	1
c.4163_4164delinsA	p.Thr1388fs	5
c.4169del	p.Leu1390fs	1
c.4223del	p.Gln1408fs	1
c.4405_4409del	p.Asp1469fs	1
c.4415_4418del	p.Lys1472fs	1
c.4478_4481del	p.Glu1493fs	10
c.4525C>T	p.Gln1509*	1
c.4631dup	p.Asn1544fs	1
c.4638del	p.Phe1546fs	1
c.4648G>T	p.Glu1550*	1
c.4712_4713del	p.Glu1571fs	2
c.4828dup	p.Val1610fs	1
c.4876_4877del	p.Asn1626fs	4
c.4889C>G	p.Ser1630*	2
c.4914dup	p.Val1639fs	1
c.4936_4939del	p.Glu1646fs	1
c.4981del	p.Tyr1661fs	1
c.5073dup	p.Trp1692fs	4
c.5116_5119del	p.Asn1706fs	3
c.5141_5144del	p.Tyr1714fs	1
c.5217_5223del	p.Tyr1739*	1
c.5217T>A	p.Tyr1739*	1
c.5279C>G	p.Ser1760*	2
c.5298del	p.Asn1766fs	1
c.5303_5304del	p.Leu1768fs	2
c.5329_5334delinsG	p.Lys1777fs	1
c.5350_5351del	p.Asn1784fs	2
c.5410_5411del	p.Val1804fs	1
c.5576_5579del	p.Ile1859fs	4
c.5641_5644del	p.Lys1881fs	1
c.5655C>A	p.Cys1885*	1
c.5682C>G	p.Tyr1894*	10
c.5722_5723del	p.Leu1908fs	2
c.5835dup	p.Ser1946fs	1
c.5857G>T	p.Glu1953*	1
c.5909C>A	p.Ser1970*	6
c.5946del	p.Ser1982fs	42

c.6049A>T	p.Lys2017*	1
c.6052_6053del	p.Ser2018*	1
c.6065C>G	p.Ser2022*	1
c.6079dup	p.Arg2027fs	1
c.6081dup	p.Glu2028fs	1
c.6275_6276del	p.Leu2092fs	27 [a]
c.6385G>T	p.Glu2129*	1
c.6405_6409del	p.Asn2135fs	1
c.6486_6489del	p.Lys2162fs	1
c.6588_6589del	p.Lys2196fs	1
c.6591_6592del	p.Glu2198fs	5
c.6602del	p.Ser2201fs	1
c.6658_6662del	p.Glu2220fs	1
c.6757_6758del	p.Leu2253fs	2
c.6829_6833del	p.Ile2278fs	1
c.6944_6947del	p.Ile2315fs	4
c.6980del	p.Leu2327*	3
c.6996_7004delins(20)	p.Cys2332fs	3
c.7008-?_7805+?del	DL	13
c.7008-?_8331+?del	DL	1
c.7069_7070del	p.Leu2357fs	5
c.7342_7343del	p.Lys2448fs	1
c.7480C>T	p.Arg2494*	4
c.7495C>T	p.Gln2499*	1
c.7543dup	p.Thr2515fs	1
c.7558C>T	p.Arg2520*	1
c.7757G>A	p.Trp2586*	6
c.7758G>A	p.Trp2586*	2
c.7762_7764delinsTT	p.Ile2588fs	4
c.7795G>T	p.Glu2599*	1
c.7884dup	p.Trp2629fs	3
c.7934del	p.Arg2645fs	1
c.7958T>C	p.Leu2653Pro	1
c.7977-1G>C	S	4
c.7977-2_-3del	S	1
c.7988A>T	p.Glu2663Val	2
c.8113dup	p.Ser2705fs	1
c.8167G>C	p.Asp2723His	6
c.8247_8248del	p.Lys2750fs	2
c.8297del	p.Thr2766fs	9
c.8395del	p.Arg2799fs	1
c.8575del	p.Gln2859fs	9
c.8633-?_8754+?del	DL	1
c.8633-?_9256+?del	DL	2
c.8756del	p.Gly2919fs	3
c.8878C>T	p.Gln2960*	1

c.8904del	p.Val2969fs	9
c.8945_8946del	p.Lys2982fs	1
c.8951C>G	p.Ser2984*	3
c.8956dup	p.Ile2986fs	1
c.9054_9055del	p.Ser3018fs	4
c.9069_9076del	p.Asn3024fs	2
c.9097dup	p.Thr3033fs	2
c.9117+1G>A	S	1
c.9117G>A	p.Val2985fs	2
c.9157del	p.Glu3053fs	2
c.9253dup	p.Thr3085fs	2
c.9257-2A>G	S	1
c.9294C>G	p.Tyr3098*	6
c.9357_9360del	p.Ile3120fs	2
c.9380G>A	p.Trp3127*	1
c.9382C>T	p.Arg3128*	7
c.9481A>T	p.Lys3161*	1
c.9490_9491del	p.Asn3164fs	1
c.9502-2A>C	S	1

DL = large deletion; S = splice site.

The pathogenic variants are specified using HGVS nomenclature (http://varnomen.hgvs.org/), using cDNA reference sequence NM_000059.3 and reference genome hg18.

[a] One participant carried both c.658_659del and c.6275_6276del.

Supplementary Table S2 – Prostate cancer risk by location of *BRCA2* pathogenic variant: adjustments and sensitivity analyses

PV location	HR (95% CI)	HR adjusted for family history [a] (95% CI)	HR adjusted for geographical location [b] (95% CI)	HR omitting the first 6 mo of follow-up (95% CI)	HR omitting related participants [c] (95% CI)	HR omitting carriers of PVs in c.756 to c.1000 [d] (95% CI)	HR omitting missense variant carriers [e] (95% CI)	HR omitting Ashkenazi founder PV carriers [f] (95% CI)
Compared with non-PCCR PVs								
Non-PCCR (5′ to c.7913)	Reference	Reference	Reference	Reference	Reference	Reference	Reference	Reference
PCCR (c.7914 to 3′)	2.34 (1.09–5.03)	2.03 (0.89–4.61)	2.50 (1.15–5.46)	2.23 (0.93–5.37)	2.93 (1.23–6.97)	2.33 (1.09–5.01)	2.56 (1.17–5.59)	2.05 (0.94–4.48)
Compared with OCCR PVs								
5′ to c.2830	1.72 (0.50–5.94)	1.77 (0.53–5.98)	1.86 (0.47–7.36)	2.55 (0.63–10.3)	1.60 (0.34–7.49)	1.75 (0.51–6.03)	1.77 (0.51–6.17)	1.49 (0.40–5.62)
OCCR (c.2831 to c.6401)	Reference	Reference	Reference	Reference	Reference	Reference	Reference	Reference
c.6402 to c.7913	3.23 (0.79–13.2)	2.86 (0.65–12.7)	4.18 (0.96–18.2)	4.03 (0.75–21.7)	3.65 (0.67–19.7)	3.24 (0.80–13.2)	3.21 (0.79–13.0)	2.77 (0.61–12.5)
PCCR (c.7914 to 3′)	3.41 (1.27–9.16)	3.00 (1.06–8.54)	3.79 (1.41–10.2)	3.96 (1.18–13.3)	4.29 (1.30–14.2)	3.42 (1.27–9.18)	3.76 (1.36–10.4)	2.89 (0.98–8.53)
Indeterminable								

CI = confidence interval; HR = hazard ratio; OCCR = ovarian cancer cluster region; PCCR = prostate cancer cluster region; PV = pathogenic variant.

[a] Number of first- and second-degree relatives diagnosed with prostate cancer.
[b] Location of recruiting clinic: London; South or East England; Wales, English Midlands or North England; Scotland or Ireland.
[c] Participants for which at least one male relative was included in the study (n = 94 omitted).
[d] Carriers of PVs in the *BRCA2* region c.756 to c.1000 suggested to be associated with increased prostate cancer risks by Patel et al [1] (n = 1 omitted).
[e] Carriers of pathogenic missense variants (n = 9 omitted).
[f] Carriers of c.5946delT (n = 42 omitted).

References

[1] Patel VL, Busch EL, Friebel TM, et al. Association of genomic domains in BRCA1 and BRCA2 with prostate cancer risk and aggressiveness. Cancer Res 2020;80:624–638.
